# Synchronous Ascending Colon Adenocarcinoma and Terminal Ileal Carcinoid Tumor Presenting With Ileocecal Intussusception: A Case Report

**DOI:** 10.7759/cureus.89883

**Published:** 2025-08-12

**Authors:** Samantha L Irizarry, Adebayo D Adunbarin

**Affiliations:** 1 Medicine, Rutgers New Jersey Medical School, Newark, USA; 2 Surgery, RWJBarnabas Health, Jersey City, USA

**Keywords:** adenocarcinoma of colon, carcinoid tumor, exploratory laparotomy, ileocecal intussusception, right-sided hemicolectomy

## Abstract

Synchronous occurrences of colorectal adenocarcinoma and gastrointestinal carcinoid tumors are rare, presenting unique diagnostic and therapeutic challenges. This report describes an 81-year-old female who developed ileocecal intussusception secondary to concurrent cecal adenocarcinoma and a carcinoid tumor in the terminal ileum. The patient underwent a right hemicolectomy with primary anastomosis, and histopathology confirmed an intramucosal adenocarcinoma within a giant tubular adenoma and a separate carcinoid tumor. Postoperative recovery was uneventful, with subsequent colonoscopy revealing benign polyps. This report highlights the importance of thorough preoperative assessment, surgical management, and vigilant postoperative surveillance in patients with synchronous malignancies.

## Introduction

Adenocarcinoma represents the most common histologic subtype of colorectal cancer, which continues to rank as one of the primary cancer death causes across the world [1,2]. Gastrointestinal neuroendocrine tumors (GINETs), also known as carcinoid tumors, are rare neoplasms with an overall reported incidence of up to 6.98 per 100,000 individuals. While they predominantly arise within the gastrointestinal tract, particularly the terminal ileum, the exact incidence at this specific site remains unclear [3,4]. These two distinct cancers manifesting together represent a rare clinical case with unknown pathophysiological roots [5-7]. Colorectal adenocarcinomas develop through the adenoma-carcinoma sequence beginning with adenomatous polyps, while carcinoid tumors emerge from enterochromaffin cells and typically show slow growth with indolent behavior [8]. The simultaneous development of these malignancies prompts investigation into common molecular pathways and genetic predispositions alongside environmental factors that may cause their concurrent appearance.

Adult intussusception occurs infrequently and is commonly associated with an underlying pathological lead point, usually a malignancy [9,10]. Unlike pediatric intussusception, which is often idiopathic, adult cases frequently necessitate surgical intervention due to the high likelihood of an obstructing lesion [11,12]. Intussusception represents approximately 1% of small bowel obstructions in adults and is found in less than one in 1,300 abdominal operations [13]. In this case, this patient's ileocecal intussusception resulted from a cecal adenocarcinoma, which underscores the importance of fast diagnostic assessment followed by surgical intervention.

This report presents a case of an 81-year-old female with ileocecal intussusception secondary to synchronous cecal adenocarcinoma and a terminal ileum carcinoid tumor, highlighting the clinical presentation, surgical approach, histopathological findings, and the importance of long-term surveillance in patients with dual malignancies.

## Case presentation

An 81-year-old female with a medical history including hypertension, hyperlipidemia, diabetes mellitus, and chronic kidney disease presented with a two-week history of lower abdominal pain and six months of diarrhea. She reported multiple episodes of watery stools daily, accompanied by occasional nausea and a reduced appetite. The patient denied recent weight loss but noted a single episode of melena the day prior to her admission. The patient was unable to recall the date or the results of her most recent colonoscopy.

Upon physical examination, she was hemodynamically stable but exhibited mild tenderness in the right lower quadrant without any peritoneal signs. Laboratory testing revealed a hemoglobin level of 10.9 g/dL and a creatinine level of 2.51 mg/dL, with an estimated glomerular filtration rate (eGFR) of 18.8 mL/min/1.73 m².

**Figure 1:**
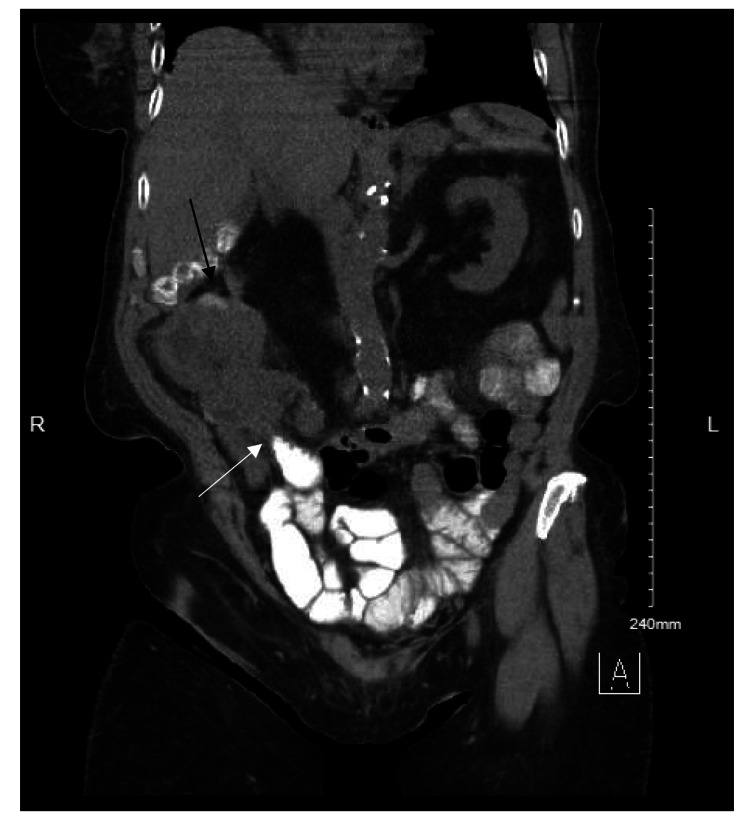
Coronal CT scan of the abdomen demonstrating ileocecal intussusception (black arrow). Notably, oral contrast appears to be stagnant at the site of intussusception (white arrow), unable to pass beyond the obstructed segment, further confirming the mechanical obstruction.

**Figure 2:**
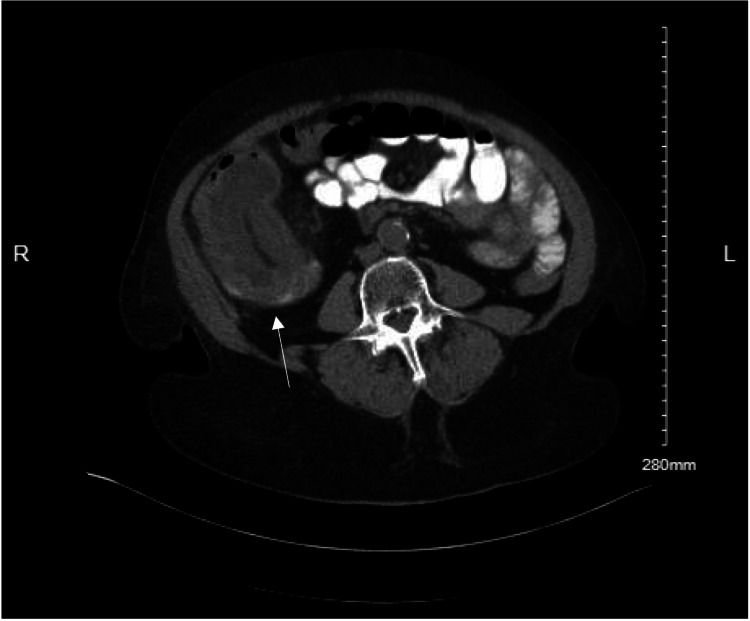
Axial CT scan of the abdomen illustrating the cross-sectional view of the ileocecal intussusception (white arrow). Cecal mass is evident along with surrounding mesenteric fat and vascular structures entrapped within the intussusceptum.

A computed tomography (CT) scan of the abdomen and pelvis with oral contrast indicated ileocecal intussusception, alongside cecal wall thickening suggestive of an underlying mass. Adjacent bowel loops showed moderate dilation, indicating partial obstruction (Figures 1, 2). Given the suspicion of malignancy and the presence of partial intestinal obstruction, the patient was scheduled for exploratory laparotomy. Due to the presenting symptoms of obstruction, signs of acute bowel obstruction based on the CT scan, the patient's age, and the likelihood of a longer time under anesthesia, the decision was made to perform an open approach.

**Figure 3:**
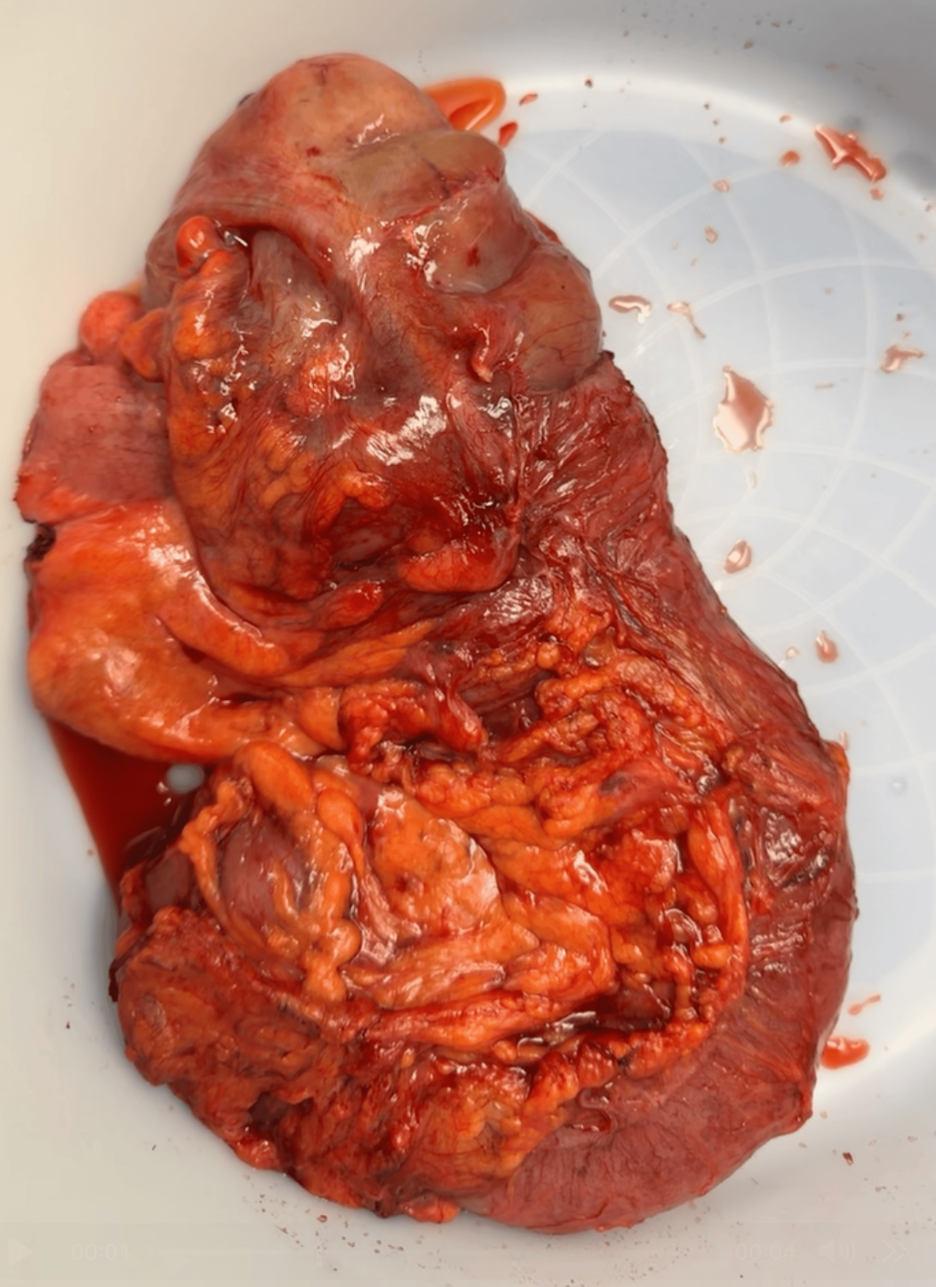
Gross pathology specimen of the resected right hemicolectomy. Exophytic, ulcerated cecal mass with overlying irregular and thickened mucosa is noted. Distal portion of the specimen reveals edematous and congested bowel. Surrounding adipose tissue and mesentery are also visible for resection margins.

During the surgical procedure, an ileocecal intussusception was identified, characterized by significant thickening of the cecal wall with a phlegmonous mass. The affected segment was resected via a right-sided hemicolectomy. This was followed by an ileocolic side-to-side anastomosis using a blue load gastrointestinal anastomosis (GIA) stapler. The common channel was then closed in two layers: the first with 2-0 Vicryl in a Connell fashion, and the second with 2-0 silk using interrupted Lembert sutures. An ostomy was not required. The specimen was removed with adequate resection of the mesentery (Figure 3).

**Figure 4:**
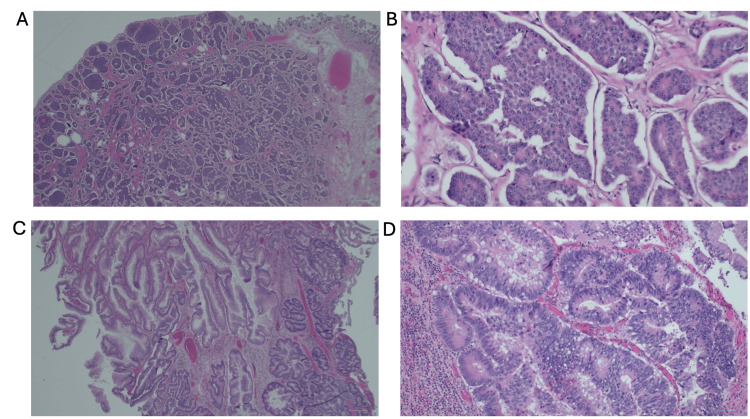
Histopathologic examination of synchronous terminal ileal carcinoid tumor and cecal adenocarcinoma (H&E stain; scale bar = 100 pixels for all images). (A, B) Terminal ileum carcinoid tumor. (A) Low-power view showing a submucosal proliferation of monotonous cells with a nested and trabecular growth pattern. (B) High-power view demonstrates classic features of a well-differentiated neuroendocrine tumor, including uniform round nuclei with finely stippled ("salt and pepper") chromatin. (C, D) Cecal adenocarcinoma. (C) Low-power view of a tubular adenoma with complex glandular architecture. (D) High-power view reveals intramucosal adenocarcinoma with stratified, hyperchromatic nuclei, loss of polarity, and crowding of dysplastic glands within the background adenomatous tissue.

Postoperative histopathological examination of the resected specimen confirmed the presence of an intramucosal adenocarcinoma arising from a giant tubular adenoma measuring 12×10×2 cm in the ascending colon (Figures 4A, 4B). This malignancy was confined to the mucosa, with no evidence of deeper invasion, and all 22 examined regional lymph nodes were negative for metastasis. Furthermore, a separate carcinoid tumor, measuring 0.7×0.5×0.5 cm, was identified in the terminal ileum, limited to the mucosa and submucosa with negative margins (Figures 4C, 4D).

Postoperative course and follow-up

The patient recovered uneventfully, demonstrating prompt return of bowel function and the ability to tolerate oral intake by postoperative day five. She was subsequently discharged with recommendations for ongoing oncologic follow-up. Given the absence of invasive adenocarcinoma and the presence of negative surgical margins, the decision was made not to pursue adjuvant chemotherapy. Several weeks after her surgery, the patient underwent a colonoscopy, during which multiple polyps were identified and subsequently removed. Histopathological examination confirmed that these polyps were benign, and no residual malignancy was detected. A follow-up CT scan was advised for six months, as well as a colonoscopy in three years, to ensure continued surveillance. The patient's baseline carcinoembryonic antigen (CEA) was 3.3 ng/mL, already within normal limits, though it was still recommended to measure at future follow-ups.

This follow-up protocol underscores the importance of monitoring patients postsurgery for signs of recurrence or the development of additional neoplasms, especially in older patients, where colorectal cancers and carcinoid tumors may occur simultaneously. Regular surveillance can aid in early detection and management of any new lesions that might arise.

## Discussion

The simultaneous appearance of adenocarcinoma and carcinoid tumors in the gastrointestinal tract constitutes a rare clinical observation [5-7]. The synchronous occurrence of adenocarcinoma in patients who have carcinoid tumors falls between 17% and 53%, yet most of these cases show that patient outcomes are primarily affected by the adenocarcinoma rather than the carcinoid tumor [7]. Carcinoid tumors accounted for nearly 0.66% of all malignant cases in extensive epidemiological studies, yet clinical outcomes change substantially when an adenocarcinoma develops concurrently due to the heightened aggression of adenocarcinomas [14].

Intussusception in adults is a rare phenomenon, accounting for only 5% of all intussusception cases and typically associated with an underlying pathology, such as malignancy, as seen in this patient [11]. In this case, the patient’s ileocecal intussusception was caused by the cecal adenocarcinoma acting as a lead point, necessitating definitive surgical resection. Recognition of this uncommon presentation is critical, as failure to promptly diagnose and intervene can result in bowel ischemia, necrosis, or perforation.

The management of adult intussusception involves careful preoperative evaluation to assess the underlying cause. Imaging modalities, such as CT scans, play a pivotal role in identifying the presence of a lead point and guiding surgical decision-making. Given the malignant etiology in most adult cases, en bloc resection rather than simple reduction is generally recommended to reduce the risk of malignant cell seeding within the luminal space [15]. In this patient, a right hemicolectomy with ileocolic anastomosis was performed, ensuring complete removal of the neoplastic lesions while maintaining bowel continuity. Studies recommend clinicians to maintain thorough surveillance and follow-up protocols for carcinoid cases due to their multicentric nature and submucosal positioning, which necessitates enhanced vigilance for identifying secondary primary tumors [16,17].

Recent genetic research reveals potential common etiological mechanisms that could make patients more susceptible to developing both adenocarcinomas and neuroendocrine tumors of the colon [18]. The underlying disease mechanisms might involve genetic predispositions related to neuroendocrine elements that release growth-stimulating substances and/or possible deficiencies in immune system monitoring that prevent tumor development and progression. Mutations and instability in multiple pathways, such as APC gene alterations along with microsatellite instability and chromosomal instability, may lead to a heightened risk of developing synchronous cancers [19].

A structured follow-up plan that integrates regular endoscopic evaluations and imaging helps manage risks and detect tumor recurrence or the development of new tumors in elderly patients. The combination of surgical intervention with regular imaging reviews and biochemical monitoring leads to superior patient management and results when treating dual malignancies [20].

## Conclusions

This case illustrates a rare but clinically significant presentation of synchronous cecal adenocarcinoma and terminal ileum carcinoid tumor resulting in ileocecal intussusception - an unusual etiology for bowel obstruction in adults. It reinforces the importance of maintaining a broad differential diagnosis when evaluating nonspecific gastrointestinal symptoms in elderly patients, especially given the increasing incidence of colorectal cancer in elderly patients. The patient’s successful right hemicolectomy with ileocolic anastomosis underscores the value of prompt diagnostic imaging, early surgical consultation, and pathology-guided management. Moreover, the incidental discovery of dual malignancies highlights the need for meticulous intraoperative and histopathological evaluation. This report adds to the limited literature on synchronous gastrointestinal tumors and underscores the importance of vigilant long-term surveillance in this patient population.
